# Botulinum Toxin Injections and Electrical Stimulation for Spastic Paresis Improve Active Hand Function Following Stroke

**DOI:** 10.3390/toxins10110426

**Published:** 2018-10-25

**Authors:** Jong-Min Lee, Jean-Michel Gracies, Si-Bog Park, Kyu Hoon Lee, Ji Yeong Lee, Joon-Ho Shin

**Affiliations:** 1 Department of NeuroRehabilitation, National Rehabilitation Center, Ministry of Health and Welfare, 58 Samgaksan-ro, Gangbuk-gu, Seoul 01022, Korea; ljm21c2000@naver.com (J.-M.L.); wldud3575@hanmail.net (J.Y.L.); 2 Service de Rééducation Neurolocomotrice, Albert Chenevier-Henri Mondor Hospital, 94000 Créteil, France; jean-michel.gracies@aphp.fr; 3 Department of Rehabilitation Medicine, Hanyang University College of Medicine, Seoul 04763, Korea; sibopark@hanyang.ac.kr (S.-B.P.); dumitru1@hanyang.ac.kr (K.H.L.)

**Keywords:** spasticity, spastic paresis, botulinum toxin, electrical stimulation, stroke management, rehabilitation, hand

## Abstract

Botulinum toxin type A (BTX-A) injections improve muscle tone and range of motion (ROM) among stroke patients with upper limb spasticity. However, the efficacy of BTX-A injections for improving active function is unclear. We aimed to determine whether BTX-A injections with electrical stimulation (ES) of hand muscles could improve active hand function (AHF) among chronic stroke patients. Our open-label, pilot study included 15 chronic stroke patients. Two weeks after BTX-A injections into the finger and/or wrist flexors, ES of finger extensors was performed while wearing a wrist brace for 4 weeks (5 days per week; 30-min sessions). Various outcomes were assessed at baseline, immediately before BTX-A injections, and 2 and 6 weeks after BTX-A injections. After the intervention, we noted significant improvements in Box and Block test results, Action Research Arm Test results, the number of repeated finger flexions/extensions, which reflect AHF, and flexor spasticity. Moreover, significant improvements in active ROM of wrist extension values were accompanied by marginally significant changes in Medical Research Council wrist extensor and active ROM of wrist flexion values. In conclusion, BTX-A injections into the finger and/or wrist flexors followed by ES of finger extensors improve AHF among chronic stroke patients.

## 1. Introduction

Upper limb spasticity (ULS) is a common impairment after stroke, which can cause abnormal posture, pain, reduced movement, and functional impairment [1,2,3,4]. Various interventions, including occupational therapy, physical therapy, oral medications, orthoses, and focal neurolysis, have been used in the management of ULS.

Intramuscular injection of botulinum toxin type A (BTX-A) is the recommended first-line treatment for focal ULS [5,6,7,8,9]. Botulinum toxin type A, which is produced by anaerobic, gram-positive, spore-forming microorganisms from the genus *Clostridium*, is the most potent toxin among various serotypes, and its safety is well established [10,11]. Its neurospecific activity, reversibility, and limited diffusion on local injection make it a safe and successful agent for symptoms characterized by hyperfunction of peripheral nerve terminals [12,13]. Although BTX-A injections have been shown to improve muscle tone, range of motion, and physical global assessment findings, evidence regarding their efficacy for improving active upper limb function (e.g., moving/manipulating objects) is lacking [14,15]. Indeed, systematic reviews have demonstrated that BTX-A has greater effects on passive function than on active function, and some studies have failed to show any improvements in active function with BTX-A treatment [4,16]. These results might hinder active treatment with BTX-A, because the ultimate goal of rehabilitation is functional improvement.

Several explanations for the discrepancy between impairment and function have been proposed. First, most previous treatments have targeted spasticity per se, while factors other than hypertonicity are likely to be associated with active function, including stretch-sensitive paresis, overactivity of agonistic muscles, and learned non-use [2,3,15]. Second, optimal adjunct therapies following BTX-A injections have not been used, despite the need for adjuvant therapy to improve efficacy [17,18]. The combination of modified constraint-induced movement therapy or active task-specific training with BTX-A injections has been shown to improve upper limb function among patients with chronic spastic hemiparesis [19,20]. However, these approaches are not available for individuals with moderate-to-severe muscle weakness, prompting the need for commonly or easily applicable optimal adjunctive therapies to augment hand function. Third, although hand function is critical for upper limb function as an end-effector, few studies have targeted active function of the hand itself [21]. As hand spasticity is particularly disruptive, improvements in shoulder or elbow function may not be adequate to ensure overall functional improvement [22]. In addition, appropriate outcome measures related to both impairment and function, especially active hand function, have not been used [23,24,25]. Although previous studies have used various outcome measures for upper limb function (e.g., disability assessment scale, motor activity log, and global self-assessment), none have focused on active hand function [20,24,26]. Fourth, standardized techniques for administering BTX-A are yet to be established, and thus there are variations in muscle selection, injection methods, and medication doses.

Many patients having ULS exhibit difficulty with finger extension, which is critical for gripping, grasping, and releasing movements. Thus, we hypothesized that relief of finger flexor spasticity following BTX-A treatment would help patients regain finger extensor strength, which would in turn improve hand function. Based on the findings of previous studies, we further hypothesized that electrical stimulation (ES) is a simple yet effective form of adjuvant therapy, as it facilitates muscle reeducation, including antagonist strengthening and agonist lengthening [27,28]. Therefore, in the present preliminary study, we aimed to determine whether BTX-A injections targeting finger flexor spasticity with concomitant ES of finger extensors can improve active hand function and impairment among patients with chronic stroke.

## 2. Results

Among the 15 included patients, 7 experienced cerebral infarction and 8 experienced intracerebral hemorrhage. Thirteen patients experienced lesions in the right hemisphere. The mean time from stroke was 12.8 ± 3.7 months. All 15 patients completed the study, and none experienced adverse events.

### 2.1. Primary Outcomes

After the intervention, there were significant improvements in Box and Block (BB) (*χ*^2^ = 6.50, *p* = 0.039), Action Research Arm Test (ARAT)-total (*χ*^2^ = 6.29, *p* = 0.043), and ARAT-gross movement scores (*χ*^2^ = 8.64, *p* = 0.013), which reflect active hand function (Table 1). Post-hoc analysis confirmed that there were significant improvements in these outcomes in the T1–T3 phase. In addition, significant improvements in ARAT-total and ARAT-gross movement scores were observed in the T2–T3 and T1–T2 phases, respectively.

### 2.2. Secondary Outcomes

Repeat-finger extensor/extension (FE) values showed significant increases (*χ*^2^ = 10.00, *p* = 0.007) in the T1–T3 and T2–T3 phases. Active-FE values showed marginally significant increases during the T2–T3 phase (*χ*^2^ = 5.615, *p* = 0.060).

Flexor spasticity significantly decreased (Modified Ashworth Scale for wrist flexor/flexion (MAS-WF): *χ*^2^ = 8.40, *p* = 0.015; MAS for finger flexor/flexion (MAS-FF): *χ*^2^ = 20.18, *p* < 0.001), and post-hoc tests revealed significant differences in the T1–T2 and T1–T3 phases, but not in the T2–T3 phase (Table 2). Significant increases in active range of motion for wrist extensor/extension (AROM-WE) values (*χ*^2^ = 9.08, *p* = 0.011) were accompanied by marginally significant increases in Medical Research Council WE (MRC-WE) (*χ*^2^ = 6.00, *p* = 0.05) and AROM-WF (*χ*^2^ = 6.00, *p* = 0.05) values. Post-hoc analyses demonstrated that AROM-WE values were higher at T3 than at T1 or T2 and that MRC-WE values significantly increased from T1 to T3. No significant changes were noted for other secondary outcomes, such as the Quick Disabilities of Arm, Shoulder, and Hand (QDASH) score (*χ*^2^ = 3.636, *p* = 0.162).

## 3. Discussion

In the present study, we attempted to examine the efficacy of BTX-A treatment targeting finger flexor spasticity and concomitant ES for improving active hand function in the chronic phase of stroke. We found that BTX-A injections into the flexor muscles of the fingers and wrist followed by ES improved active hand function, as indicated by significant improvements in BB, ARAT (ARAT-total, ARAT-gross movement), and Repeat-FE scores. These findings are in contrast to those of previous studies, which failed to demonstrate improvements in active function after BTX-A treatment among patients with chronic stroke [29,30,31].

These discrepancies may be explained by our concept of intervention, as we considered treatment according to spastic paresis rather than flexor muscle spasticity only [2,3]. Overactivity of flexor muscles due to spasticity impedes finger and wrist extension, aggravating stretch-sensitive paresis and inhibiting voluntary recruitment of extensors. Similarly, a previous study demonstrated co-activation of spastic flexors and weak extensors [32]. Rehabilitation strategies often focus on finger/wrist flexors, as most hand functions are related to flexor movement (e.g., grasping, pinching, and manipulating objects). However, many patients with ULS following stroke exhibit difficulty with finger extension, which is needed to initiate hand grip and grasp. Thus, we attempted to restore the balance between the flexors and extensors of the wrist/fingers by targeting asymmetrical spastic paresis in two main steps (BTX-A injections and ES with a wrist brace).

Botulinum toxin type A injections to finger/wrist flexors reduced involuntary activation of the flexor muscles and further led to “therapeutic weakness” [33]. However, such weakness is only observed when the doses of BTX-A administered at the flexor digitorum superficialis and profundus are much larger than the doses recommended by the current consensus report [34,35]. Therapeutic weakening of the agonistic finger/wrist flexors allowed for restoration of antagonistic finger/wrist extensor function by reducing reciprocal inhibition from the flexors, stretch-induced paresis, agonistic flexor overactivity, and reflex tone [2,3,36].

Improved balance between agonist and antagonist muscles was accomplished with proprioceptive discharge reduction via group-IA afferents [37,38]. Botulinum toxin type A decreased afferent input of the muscle spindles and their stretch sensitivity. Presynaptic acetylcholine release block with BTX-A was more profound and earlier in gamma-motoneurons than in alpha-motoneurons. From the viewpoint of cortical activity, this BTX-A effect on proprioception is important because abnormal spindle activity generating irregular proprioceptive input may result in abnormal intracortical inhibition and cortical excitability [39]. Similarly, BTX-A-induced spasticity relief was associated with central sensorimotor activation change, which was likely mediated by an altered afferent input from spastic muscles [40]. Moreover, BTX-A reduced the activation of the contralateral putamen and thalamus via modulation of basal ganglia activation [41].

On the other hand, fascicle selection was tailored to each patient, and a flexible dosing regimen was utilized to ensure the appropriate level of weakness without inadvertent over-weakness. This may explain why we observed significant changes in ARAT-gross movement and MAS-WF/finger flexor/flexion (FF) values, but not MRC-WF/FF values or functional deterioration, between T1 and T2.

We subsequently utilized ES to facilitate target muscle (finger/wrist extensor) contraction, which has been difficult to achieve owing to increased flexor tone. ES enabled reeducation, stretching, and strengthening of the extensor muscles [28]. This selective facilitation of agonistic extensor muscles resulted in significant improvements in AROM-WE between T2 and T3, rather than improvements in AROM-WF. Concordantly, active function-related improvements were observed in ARAT-total, ARAT-gross movement, Repeat-FE, and Active-FE values during this phase, but not between T1 and T2.

Such differences between T1–T2 and T2–T3 may explain the discrepancies between active and passive functions following BTX-A treatment [42]. Our findings suggest that spasticity control is insufficient for restoring active function and that concomitant interventions selectively facilitating agonist muscles are necessary to improve outcomes. Notably, cyclic ES of both finger flexors and extensors following BTX-A injections has not been shown to be associated with functional improvements [25], suggesting that such interventions should target agonist function rather than antagonist spasticity per se.

In addition, we utilized a wrist brace to maximize finger extension during ES while reducing unwanted wrist or metacarpophalangeal joint hyperextension. Full finger extension is difficult during concomitant wrist extension owing to passive tension that results from the muscle insertion pattern of two-joint finger flexor muscles. The wrist brace maintained the wrist/metacarpophalangeal joints in a neutral position to allow sufficient extension of the distal or proximal interphalangeal joints, and it may have decreased stretch-sensitive paresis of the finger extensors.

In the present study, we attempted to utilize comprehensive hand-relevant outcome measures for both impairment (MRC, MAS, and AROM) and active function (ARAT and BB). We also utilized measures specific to hand function, particularly finger extension (Active FE, Distance-FP, Repeat-FE, and grip strength). These various measures assessed at different time points helped classify the effects of our intervention in terms of spastic paresis. Recently, goal attainment scales have been used to determine individualized, focal changes following BTX-A injections [43]. However, such scales do not measure objective outcomes but rather measure subjective achievement according to the opinions of the physicians and patients [44]. Therefore, we believe that the objective scales utilized in the present study are more appropriate than goal attainment scales for determining focal functional changes in clinical trials.

According to the QDASH results, no improvement in subjective upper limb function was observed, and this is likely due to the small yet significant objective change over the course of the intervention. Further studies should examine whether both subjective and objective improvements in upper limb function can be observed in select groups of patients, such as those in the subacute phase of stroke and those with some level of strength in the wrist/finger extensors.

The present study had several limitations. First, as this was not a randomized controlled trial, it is difficult to conclude whether our intervention would outperform other interventions or whether the combination of BTX-A injections and ES is more effective than either approach alone. Second, we did not measure outcomes at 12 weeks, when the effects of BTX-A can no longer be observed [45]. Finally, we did not take into account thumb function. Considering that thumb function accounts for 40% of hand performance, future studies should investigate the efficacy of our intervention with regard to thumb function [46].

## 4. Conclusions

Our findings indicate that BTX-A injections into the finger and/or wrist flexor muscles followed by ES of finger extensors improve active hand function and impairment among patients with chronic stroke. The present results support the notion that interventions targeting spastic paresis, rather than spasticity per se, may improve active hand function.

## 5. Materials and Methods

The present open-label, pilot study included 15 patients (14 men; 14 right-handed individuals; mean age, 44.7 ± 15.0 years; age range, 22–74 years; Table 3) from the stroke unit of a rehabilitation hospital between March 2016 and February 2017. Patients engaged in inpatient rehabilitation programs at the time of enrollment were allowed to continue therapy throughout the intervention, and anti-spasticity medications were continued at current or reduced doses or were discontinued. The study was approved by the ethics review board of our rehabilitation hospital (NRC-2016-02-011), and all participants provided written informed consent prior to enrollment. The study was registered with ClinicalTrials.gov (number NCT 03549975, 29 March 2016).

### 5.1. Inclusion and Exclusion Criteria

The inclusion criteria were as follows: (1) age older than 18 years; (2) hemiplegic ULS secondary to unilateral ischemic or hemorrhagic stroke; (3) finger and wrist flexor spasticity graded at least 1+ on the MAS; and (4) stroke occurrence at least 6 months prior to enrollment. The exclusion criteria were as follows: (1) fixed contracture of the wrist or hand; (2) previous ULS treatment with neurolytic therapy, surgery, or BTX-A injection; (3) presence of any active implanted device; (4) presence of any neurological disorder causing motor deficit or spasticity, other than stroke; (5) inability to cooperate for all outcome measure-related tasks secondary to cognitive impairment or aphasia; (6) pregnancy, planned pregnancy, or lactation; and (7) contraindication for BTX-A treatment.

### 5.2. Intervention

#### 5.2.1. BTX-A Administration

Patients received BTX-A (onabotulinumtoxin A, Botox; Allergan Inc., Irvine, KY, USA) injections at week 0. The dilution of BTX-A was standardized, such that each vial of BTX-A (100 U) was diluted with 2 mL of normal saline (5 U/0.1 mL). A physician with experience in BTX-A treatment performed clinical evaluations of ULS and freely selected the target muscle and dose according to the pattern and severity of ULS in each patient. BTX-A injections were then administered under ultrasonographic guidance (ACCUVIX XG; Medison, Seoul, Korea). Injections at the flexor digitorum superficialis and profundus were administered by tailoring fascicle selection to each patient and by adopting a flexible dosing regimen. In each patient, the total BTX-A dose did not exceed 360 U. The dosages and injection sites are listed in Table 3.

#### 5.2.2. ES with a Wrist Brace

After 2 weeks of BTX-A injections, patients received ES of finger extensors while wearing a wrist brace for 4 weeks. ES was administered 5 days per week during 30-min sessions by trained occupational therapists, using a Novastim CU-FS1 unit (CU, Medical, Seoul, Korea). During ES, each patient was seated in a chair with the affected arm resting on a table and with a small pillow supporting the pronated forearm. Cyclic ES was administered to induce muscle contractions, using biphasic pulses (frequency, 50 Hz; pulse duration, 200 μs) delivered for 6 s (ramp up, 1 s; ramp down, 2 s), with a 12-s burst-off. Two surface electrodes (5 × 5 cm) were placed over the extensor digitorum communis. The amplitude of stimulation was adjusted to elicit adequate finger extension for a sufficient range of motion against gravity, without pain.

The wrist brace (National Rehabilitation Center, Seoul, Korea) used during ES was made of soft fabric, and it covered the area from the wrist to the metacarpophalangeal joint. Two metal bars were attached to the volar and dorsal regions of the brace to immobilize the wrist in a neutral position. This immobilization ensured that ES could induce selective distal or proximal interphalangeal joint extension while overcoming unwanted simultaneous wrist or metacarpophalangeal joint extension. A Velcro strap was used to restrain the dorsal and volar sides of the brace.

### 5.3. Outcome Measures

**Figure 1:**
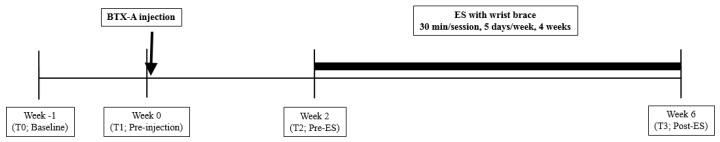
Experimental design. Primary and secondary outcomes are assessed at baseline (week 1, T0), immediately before BTX-A injections (week 0, T1), and 2 weeks (week 2, T2) and 6 weeks (week 6, T3) after BTX-A injections, by an experienced research occupational therapist. Patients exhibiting changes in outcomes between T0 and T1 were excluded. BTX-A: botulinum toxin type A, ES: electrical stimulation.

We recorded baseline characteristics, including sex, age, stroke type, brain lesion side, time since stroke, and handedness. Primary and secondary outcomes were assessed at baseline (week −1, T0), immediately before BTX-A injections (week 0, T1), and 2 weeks (week 2, T2) and 6 weeks (week 6, T3) after BTX-A injections, by an experienced research occupational therapist. For further screening, we evaluated changes in outcomes between T0 and T1. Patients exhibiting changes between these two time points were excluded from the study. We examined the effects of BTX-A at 2 weeks, as previous studies have indicated that BTX-A shows maximum efficacy between 1 and 2 weeks after treatment [47]. Outcomes at T3 represented the combined effects of BTX-A and ES with a wrist brace (Figure 1).

#### 5.3.1. Primary Outcomes

The primary outcomes were changes in patient results in the BB test and ARAT between T1 and T3, which represent changes in the active function of the upper limb following the present intervention. The BB test is a reliable and validated measure of unilateral gross manual dexterity, in which participants are required to pick up a 1-inch block, lift it over a partition, and then release it within a target area as many times as possible within 60 s [48]. The ARAT is a reliable and validated measure of arm function using observational methods with the following four subsections: grasp, grip, pinch, and gross movement. It comprises 19 items rated using a 4-point ordinal scale (0–3). Thus, the total scores on the ARAT range from 0 (failure to perform all movements; patients cannot perform any part of the test) to 57 (all movements are performed normally) [49].

#### 5.3.2. Secondary Outcomes

Hand-specific active function was assessed according to active finger extension (Active-FE), the maximum distance from the fingertip to the palm (Distance-FP), the number of repeated finger flexion/extension movements within 20 s (Repeat-FE), the rating of active thumb opposition (Thumb-opposition), and grip strength as measured using the Jamar dynamometer (Sammons Preston, Bolingbrook, IL, USA). Active-FE was rated using a 5-point ordinal scale (0 = no movement, 4 = functional extension of all digits) by reviewing a video clip [50]. Distance-FP (cm) values were obtained by measuring the distance from the middle fingertip to the mid-palmar crease [51]. Thumb-opposition was rated using a 7-point scale (1 = to the distal phalanx of the index finger, 7 = to the medial phalanx of the small finger) [51].

Upper limb impairment was assessed according to MAS scores for the wrist and finger flexors/extensors (MAS-WF/WE/FF/FE), MRC grades for wrist and finger flexor/extensor strength (MRC-WF/WE/FF/FE), and active range of motion for wrist flexion/extension (AROM-WF/WE) and radial/ulnar deviation (AROM-RD/UD) as determined through goniometric measurement. Modified Ashworth Scale grades of spasticity were converted from 1+, 2, 3, and 4 to 2, 3, 4, and 5, respectively [52].

Subjective general upper limb function was evaluated using the self-reported QDASH scale. The QDASH scale consists of 11 items regarding physical function and symptoms during the previous week, and the total scores range from 0 (no disability) to 100 (most severe disability) [53].

### 5.4. Statistical Analysis

For all outcome measures, the Friedman test was used to determine the overall change from pre-intervention (T1) to post-intervention (T2)/follow-up (T3). When the Friedman test yielded significant results, various pairwise comparisons were performed using post-hoc Wilcoxon signed-rank tests in order to determine the effect of each intervention (BTX-A/ES). All statistical analyses were performed using SPSS 20.0 (IBM SPSS Statistics, IBM Corporation, Armonk, NY, USA). The level of statistical significance was set at *p* < 0.05.

## Figures and Tables

**Table 1:** Changes in primary outcome measures over time.

Outcome					*p* ^†^		
	T1	T2	T3	*p* *	T1–T2	T2–T3	T1–T3
**BB test**	3.07 ± 3.85	3.60 ± 4.91	4.67 ± 5.25	0.039	0.473	0.120	0.028
**ARAT-total**	11.33 ± 8.03	11.27 ± 7.71	12.73 ± 7.67	0.043	1.000	0.036	0.044
**ARAT-grasp**	2.87 ± 3.82	3.00 ± 3.98	3.27 ± 3.75	0.276	0.854	0.334	0.276
**ARAT-grip**	2.13 ± 2.07	1.67 ± 1.92	2.53 ± 1.92	0.120	0.141	0.059	0.257
**ARAT-gross movement**	5.67 ± 2.16	5.93 ± 2.15	6.27 ± 2.37	0.013	0.046	0.096	0.021
**ARAT-pinch**	0.67 ± 1.23	0.67 ± 0.98	0.67 ± 0.98	1.000	1.000	1.000	1.000

Data are presented as mean ± standard deviation. Outcomes were assessed at immediately before (T1), 2 weeks (T2), and 6 weeks of BTX-A injections (T3). * Friedman test; ^†^ Post-hoc Wilcoxon signed-rank test between T1 and T2, between T2 and T3, and between T1 and T3. ARAT: Action Research Arm Test, BB: Box and Block.

**Table 2:** Changes in secondary outcome measures over time.

Outcome					*p* ^†^		
	T1	T2	T3	*p* *	T1–T2	T2–T3	T1–T3
**Active-FE**	1.73 ± 0.88	2.00 ± 0.85	2.20 ± 0.94	0.060	0.102	0.180	0.053
**Distance-FP (cm)**	2.58 ± 3.12	3.80 ± 3.02	3.67 ± 2.58	0.212	0.023	0.655	0.027
**Repeat-FE**	2.07 ± 1.58	2.27 ± 1.16	3.13 ± 1.77	0.007	0.558	0.017	0.008
**Thumb opposition**	0.07 ± 0.26	0.13 ± 0.35	0.33 ± 0.62	0.223	0.317	0.180	0.102
**MAS-WF**	2.13 ± 0.35	1.80 ± 0.41	1.73 ± 0.46	0.015	0.025	0.317	0.014
**MAS-WE**	0.20 ± 0.41	0.13 ± 0.35	0.07 ± 0.26	0.223	0.317	0.317	0.157
**MAS-FF**	2.33 ± 0.49	1.67 ± 0.49	1.60 ± 0.51	<0.001	0.002	0.317	0.001
**MAS-FE**	0.07 ± 0.26	0.00 ± 0.00	0.00 ± 0.00	0.368	0.317	1.000	0.317
**MRC-WF**	2.40 ± 0.91	2.40 ± 0.91	2.53 ± 0.83	0.135	1.000	0.157	0.157
**MRC-WE**	2.27 ± 0.88	2.40 ± 0.74	2.53 ± 0.83	0.050	0.157	0.157	0.046
**MRC-FF**	2.60 ± 0.51	2.60 ± 0.51	2.67 ± 0.49	0.717	1.000	0.317	0.564
**MRC-FE**	1.47 ± 0.64	1.60 ± 0.63	1.67 ± 0.62	0.097	0.157	0.317	0.083
**AROM-WF (°)**	46.67 ± 26.37	46.67 ± 26.37	49.33 ± 23.44	0.050	1.000	0.102	0.102
**AROM-WE (°)**	32.67 ± 26.04	33.67 ± 25.53	38.00 ± 24.55	0.011	0.396	0.024	0.023
**AROM-RD (°)**	4.67 ± 7.42	5.33 ± 6.40	6.67 ± 7.24	0.174	0.564	0.157	0.083
**AROM-UD (°)**	4.67 ± 6.40	5.33 ± 6.40	5.33 ± 6.40	0.368	0.317	1.000	0.317
**Grip strength (kg)**	0.27 ± 1.03	0.13 ± 0.52	0.33 ± 1.05	0.223	0.317	0.180	0.317
**QDASH**	56.88 ± 17.22	54.65 ± 14.88	53.87 ± 16.54	0.162	0.220	0.529	0.058

Data are presented as mean ± standard deviation. Outcomes were assessed at immediately before (T1), 2 weeks (T2), and 6 weeks of BTX-A injections (T3). * Friedman test; ^†^ Post-hoc Wilcoxon signed-rank test between T1 and T2, between T2 and T3, and between T1 and T3. FE: finger extensor/extension, FP: fingertip to the palm, MAS: Modified Ashworth Scale, WF: wrist flexor/flexion, WE: wrist extensor/extension, FF: finger flexor/flexion, MRC: Medical Research Council, AROM: active range of motion, RD: radial deviation, UD: ulnar deviation, QDASH: Quick Disabilities of Arm, Shoulder, and Hand, °: degree.

**Table 3:** Clinical characteristics of the study population.

Patient	Stroke Type	Lesion Side	Time from Stroke (Months)	Dominant Hand	Injection Site (Dosage in Units)
**1**	Ischemic	Rt.	11.5	Rt.	FCU (25), FDP 2/3/4/5 (10/20/20/10), FDS 2/3/4/5 (20/30/25/15), FPL (25)
**2**	Ischemic	Rt.	10.9	Rt.	FCU (25), FDP 2/3/4 (15/20/15), FDS 2/3/4/5 (25/25/30/15), FPL (30)
**3**	Hemorrhagic	Rt.	13.2	Rt.	FCU (40), FDP 2/3/4 (10/15/15), FDS 2/3/4/5 (25/30/35/20), FPB (10), FPL (30), PT (50)
**4**	Hemorrhagic	Rt.	17.4	Rt.	AP (10), BB (40), brachialis (50), FDP 2/3/4/5 (10/15/15/5), FDS 2/3/4/5 (25/35/40/15), FPL (40)
**5**	Ischemic	Rt.	18.9	Rt.	AP (10), brachialis (55), FDP 2/3/4 (10/10/10), FDS 2/3/4/5 (30/40/35/20), FPL (40), PT (40)
**6**	Ischemic	Rt.	10.2	Rt.	FCU (30), FDP 2/3/4/5 (10/20/10/10), FDS 2/3/4/5 (25/25/20/15), FPB (5), FPL (25), PT (20)
**7**	Hemorrhagic	Lt.	12.6	Rt.	Brachialis (60), FCU (40), FDP 2/3/4 (10/10/15), FDS 2/3/4/5 (25/35/30/15), FPL (20), PT (40)
**8**	Ischemic	Lt.	13	Rt.	Brachialis (60), deltoid (20), FDP 2/3/4/5 (15/15/10/10), FDS 2/3/4/5 (30/40/30/10), FPB (10), FPL (30), PT (20)
**9**	Hemorrhagic	Rt.	10.3	Lt.	Brachialis (60), deltoid (20), FDP 2/3/4/5 (15/15/10/10), FDS 2/3/4/5 (30/40/30/10), FPB (10), FPL (30), PT (20)
**10**	Hemorrhagic	Rt.	20.3	Rt.	AP (10), FCU (30), FDS 2/3/4 (10/15/10), FPB (10), FPL (20), lumbricals (10)
**11**	Ischemic	Rt.	8.6	Rt.	AP (10), FCU (30), FDP 2/3/4/5 (5/10/10/5), FDS 2/3/4/5 (20/30/20/10), FPL (20)
**12**	Ischemic	Rt.	7.2	Rt.	AP (10), BB (40), FDP 2/3/5 (10/10/5), FDS 2/3/4/5 (30/35/20/10), FPL (30)
**13**	Hemorrhagic	Rt.	9.8	Rt.	AP (15), FDP 2/3/4 (5/10/5), FDS 2/3/4/5 (10/20/15/5), FPL (15)
**14**	Hemorrhagic	Rt.	12.8	Rt.	FDP 2/3/4/5 (30/30/35/25), FDS 2/3/4 (10/15/15), FPL (30), opponens (5)
**15**	Hemorrhagic	Rt.	14.9	Rt.	Brachialis (50), brachioradialis (20), FCU (30), FDP 2/3/4 (15/15/15), FDS 2/3/4/5 (35/40/30/20), FPL (30)

AP: adductor pollicis, BB: biceps brachii, FCU: flexor carpi ulnaris, PT: pronator teres, FDP: flexor digitorum profundus, FDS: flexor digitorum superficialis, FPB: flexor pollicis brevis, FPL: flexor pollicis longus, Rt.: right, Lt.: left.
